# A New Approach for the Assessment of True Maxillomandibular Sagittal Relationship: A Zeta Angle

**DOI:** 10.7759/cureus.57788

**Published:** 2024-04-07

**Authors:** Nikita Mohelay, Nisha Dua, Sameena B Maqhbool, Salim Shamsuddin, Khadeer Riyaz, Vijay Sonawane

**Affiliations:** 1 Department of Orthodontics & Dentofacial Orthopedics, Manav Rachna Dental College, Manav Rachna International Institute of Research and Studies (MRIIRS), Faridabad, IND; 2 Department of Oral Medicine and Maxillofacial Radiology, Swami Devi Dyal Hospital and Dental College, Panchkula, IND; 3 Department of Orthodontics & Dentofacial Orthopedics, The Oxford Dental College and Research Centre, Bangalore, IND; 4 Department of Orthodontics & Dentofacial Orthopedics, Rural Dental College, Pravara Institute of Medical Sciences, Loni, IND

**Keywords:** novel, maxillomandibular, dysplasia, skeletal, sagittal

## Abstract

Introduction: The assessment of sagittal skeletal dysplasia is crucial for accurate diagnosis and treatment planning, thus necessitating a thorough evaluation. A novel cephalometric parameter, known as the Zeta angle, was proposed in this study for the evaluation of the maxillomandibular relationship in the sagittal plane.

Materials and methods: This observational study was carried out using 291 pre-treatment lateral cephalograms of participants aged between 15 and 25 years, categorized into skeletal class I, II, and III relationships based on Wits appraisal, ANB angle, and Beta angle. The individuals reported to the Department of Orthodontics from January 2021 to October 2023, Manav Rachna Dental College, Manav Rachna International Institute of Research and Studies, Faridabad. The Zeta angle was constructed using three skeletal reference points as point Pt (pterygoid), point M (center of premaxilla), and point Pm (suprapogonion) and was measured perpendicular to point M on the Pt-Pm line and the M-Pm line to determine the severity and type of maxillomandibular discrepancy in the sagittal plane. Statistical tests were used to obtain the mean values for the Zeta angle. Analysis of variance (ANOVA), followed by the Bonferroni post-hoc test, was used to assess skeletal differences between the groups. Receiver operating characteristic (ROC) curves were used to evaluate the sensitivity and specificity of this angle.

Results: The results indicated that a Zeta angle between 57° and 64° had a class I skeletal jaw pattern, a Zeta angle less than 57° indicated a class II skeletal jaw pattern, and a Zeta angle greater than 64° indicated a class III skeletal jaw pattern. ROC curves showed that a Zeta angle less than 57.5° had 80% sensitivity and 82.5% specificity in distinguishing class II from the class I subset. A Zeta angle greater than 64.5° has a sensitivity and specificity of 92.5% in distinguishing class III from the class I subset.

Conclusion: The Zeta angle may serve as an additional diagnostic tool for the reliable determination of sagittal jaw relationships due to its establishment based on stable anatomical points, thus remaining unaffected by jaw rotations and orthodontic treatments.

## Introduction

Accurate estimation of dentofacial discrepancy is crucial for planning and achieving optimal aesthetic results in orthodontic treatment. Cephalometric assessments have a noteworthy impact within the field of orthodontics when it comes to diagnosing and planning treatments, specifically in evaluating anteroposterior (A-P) dysplasia. Currently, numerous linear and angular measurements are available to evaluate A-P discrepancies between the maxillary and mandibular apical bases, aiding in formulating a customized treatment plan for each case [1-4]. The ANB angle is frequently utilized as a criterion, despite the presence of discrepancies in nasion movement and jaw rotations that can be influenced by growth or orthodontic interventions, consequently impacting points A (deepest point on the concavity of maxilla) and B (deepest point on the concavity of mandible) [1-3].

To address these limitations, Jacobson introduced "The Wits Appraisal" [4]. Nevertheless, it is influenced by the emergence of teeth or orthodontic interventions, thus hindering its ability to accurately diagnose pure sagittal discrepancies [5,6]. In addition, notable variations in gender and ethnicity have been observed [5]. Researchers have frequently depended on the consistency of the palate across different time points to determine linear and angular measurements [7]. However, the alteration in the palatal plane inclination due to growth and orthodontic procedures raises doubts about its reliability and complicates the determination of mean values [8]. Hence, the necessity arose for a parameter that is not influenced by occlusion or cranial reference planes to be used to evaluate apical base dysplasia. However, despite being unaffected by cranial landmarks, the Beta angle may undergo significant changes at points A and B throughout growth and as a result of orthodontic interventions. Furthermore, the identification of the center of the condyle poses a challenge [9,10].

These limitations were addressed by the Yen [11] and W angles [12]. Nevertheless, the validity of the Yen angle is questionable because of jaw rotation resulting from skeletal growth or orthodontic procedures. Conversely, while the W angle is not impacted by jaw rotations, it incorporates the center of the Sella turcica, a factor deemed unreliable by several research investigations [13].

The present study introduced a novel cephalometric parameter “Zeta angle” to assess sagittal maxillo-mandibular discrepancy. The Zeta angle is constructed on stable landmarks using three skeletal reference sites: point Pt (the point at the junction of the pterygomaxillary fissure and foramen rotundum), point M (the mid-point of the center of the premaxilla), and point Pm (the point at which the shape of the chin changes from convex to concave). The study aimed to interpret the mean values and standard deviation for the Zeta angle in populations with class I, II, and III apical base patterns.

## Materials and methods

Study design

An observational, retrospective, cross-sectional study was performed on 291 pre-treatment cephalometric radiographs of individuals who reported to the Department of Orthodontics from January 2021 to October 2023, Manav Rachna Dental College, Manav Rachna International Institute of Research and Studies, Faridabad. All individuals in the study cohort were from Northern India, and the research protocol was approved by the Institutional Review Board. Before initiating orthodontic treatment, all patients provided written informed consent by the department's protocol, allowing their clinical records to be used for research purposes.

Sample size calculation

The sample size was calculated using an expected sensitivity and specificity of 90% and 85%, respectively, a 25% prevalence rate of sagittal dysplasia, an alpha error of 5%, and a 95% confidence interval. The calculation was performed using G*Power software version 3.2.9 (Universität Kiel, Germany), and the sample size was 97 samples in each group. As the present study was conducted in three groups, the total sample size was 291.

Methodology

The lateral cephalograms of the following patients were included in the study: age of 15-25 years, average growth pattern (SN-GoGn angle of 27-36^0^) [14], no history of previous orthodontic treatment, no craniofacial anomalies, presence of all permanent teeth except third molars, and presence of good-quality lateral cephalograms where landmarks used in the study could be easily and clearly visualized.

All lateral cephalograms were obtained using a KODAC 8000 C Digital Panoramic and Cephalometric system (Carestream Health Inc., Rochester, NY, USA) in the voltage range of 70 kV and a current range of 10 mA. The patients were positioned within the cephalostat, such that the sagittal plane intersected at a perpendicular angle to the trajectory of the X-rays. The primary beam was oriented toward the left aspect of the face with a standardized level of magnification set at 10 percent, while ensuring that the Frankfort horizontal (FH) plane remained parallel to the horizontal plane. All participants were directed to occlude centric relations while ensuring that their lips were gently sealed. Each cephalogram was captured by an oral and maxillofacial radiologist with a decade of expertise. A total of 291 pre-treatment lateral cephalograms were traced manually on a 0.003” thick acetate matte tracing paper (0.3 mm, 3H mechanical lead pencil). The ANB angle [1], Wits appraisal [4], Beta angle [9], and SN-GoGn angle [14] were measured and compared by two examiners separately, and the mean values were evaluated. The ANB angle, Wits appraisal, and Beta angle were used to determine the anteroposterior discrepancy, whereas the SN-GoGn angle indicated the skeletal pattern in the vertical dimension. The details of the angles with the landmarks are provided in Table 1.

**Table 1:** Cephalometric landmarks used in the study Reference: Jacobson A [15]

Sr.no	Landmarks	Description
1	N	Nasion (the anterior-most point on the frontonasal suture)
2	A	The deepest point in the anterior concavity of the maxilla
3	B	The deepest point in the anterior concavity of the mandible
4	ANB angle	An angle constructed between N-to-A and A-to-B points
5	Po	Porion (most superior point on the external auditory meatus)
6	Or	Orbitale (most inferior point on the orbit)
7	Frankfort horizontal plane	A plane joining Po and Or
8	S	Sella (mid-point of the sella tursica)
9	Gn	Gnathion (the most anteroinferior point on the bony chin)
10	Go	Gonion (the constructed point at the intersection of the Ramal and mandibular plane)
11	SN-GoGn	Mandibular plane angle (the angle between the S-N plane and Go-Gn plane)
12	Occlusal plane	A plane passing through cusps of premolars and first molar
13	Wits appraisal	The linear distance between perpendiculars dropped from points A and B on the occlusal plane
14	C	Condylion (the most posterior-superior point on the mandibular condyle)
15	Beta angle	The angle formed between the A-B line and the perpendicular through point A from the apparent axis of the C point

Of the total 596 pre-treatment cephalograms screened, 291 lateral cephalograms were selected and subcategorized into three groups, that is, class I, II, and III skeletal jaw base, each consisting of 97 lateral cephalograms, based on pre-set inclusion and exclusion criteria. They were further segregated according to gender, with 48 males (49%) and 49 females (51%) in each group.

The criteria for segregation into skeletal class I were the presence of an ANB angle of 2°-4°, Wits appraisal of 0 to 3 mm, Beta angle of 27-35°, and a pleasant profile. The criteria for skeletal Class II were ANB angle ≥4°, Wits appraisal ≥3 mm, Beta angle<27°, and convex profile. The criteria for skeletal Class III were an ANB angle ≤2°, Wits appraisal ≤0 mm, Beta angle >35°, and a concave profile.

Zeta angle

**Figure 1:**
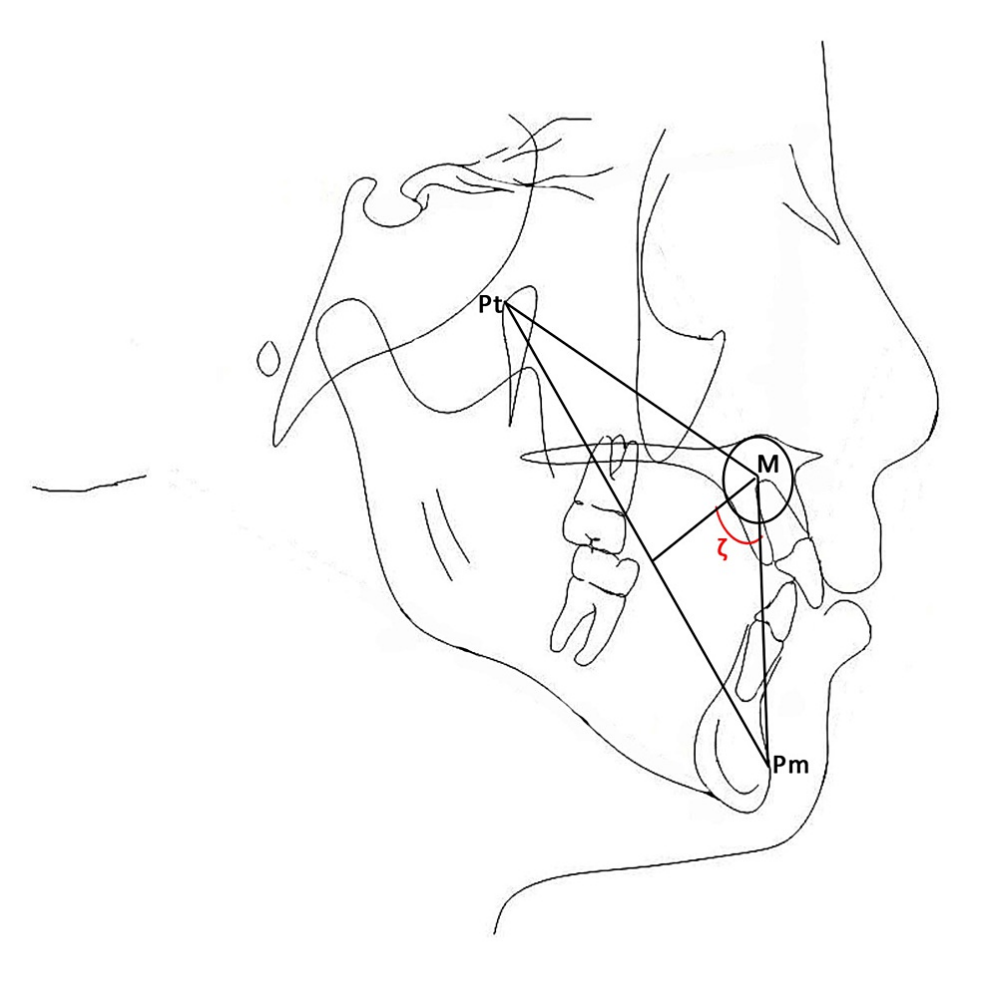
Zeta angle (ζ) and reference lines Point Pt (the junction of the pterygomaxillary fissure and foramen rotundum), point M (the center of the premaxilla), point Pm (the point at which the shape of the chin changed from convex to concave), and angle ζ (the Zeta angle between the perpendicular line and M-Pm line) Image created by the authors.

The Zeta angle introduces a novel parameter for the evaluation of the anteroposterior relationship. This criterion relies on the utilization of three anatomical landmarks, namely, point Pt, point M, and point Pm, in order to assess the magnitude and nature of the sagittal disparity between the maxillary and mandibular apical bases. The angle can be established by locating three points: Point Pt, the junction of the pterygomaxillary fissure, and foramen rotundum. The delineation of the foramen rotundum may be recognized using a specialized template intended for this specific task, such as the Jacobson-Sadowsky lip contour template by Unitek Corp. Alternatively, it can be estimated at the 10:30 position on the circular configuration of the superior border of the pterygomaxillary fissure [15]. This point is referred to as point M and denotes the midpoint of the premaxilla. The center of the premaxilla was identified using a template of concentric circles with increments of 0.5” in diameter. The center of the template was located, and point M was marked on the tracings [15]. Point Pm was marked as the point at which the shape of the chin changed from convex to concave [15]. The subsequent step involves defining three lines through the connection of specific points: the line linking point Pt to point Pm (referred to as the Pt-Pm line), the line connecting point M to point Pm (known as the M-Pm line), and the line extending from point M at a perpendicular angle to the Pt-Pm line. The Zeta angle was measured between the perpendicular line and the M-Pm line, as shown in Figure 1.

Reliability

All cephalometric measurements were independently performed by two experienced orthodontists with more than five years of experience. The measurements were replicated on 40 randomly chosen cephalograms two weeks later by identical examiners who were unaware of the cohorts and prior measurements.

Statistical analysis

The statistical analysis conducted in IBM SPSS Statistics for Windows, version 26.0 (released 2019, IBM Corp., Armonk, NY) involved tabulating data collected by two examiners in an Excel sheet. The parametric data were presented as means and standard deviations. The inter- and intra-examiner reliabilities of cephalometric measurements were assessed by calculating the intraclass correlation coefficient. The Shapiro-Wilk test was used to evaluate the skewness of the data, which confirmed a normal distribution. To assess statistically significant differences among the mean values of the three groups, an analysis of variance (ANOVA) test was employed along with the Bonferroni post-hoc test. Sex differences were determined using independent t-tests. Receiver operating characteristic (ROC) curves were used to assess the sensitivity and specificity of the angle. Statistical significance was established at P ≤ 0.05.

## Results

The intra-examiner reliability was 96%, while the inter-examiner reliability was 94%, indicating commendable reliability and reproducibility. The mean Zeta angle for the class I skeletal base group was recorded at 60.2° ± 3°. Conversely, the mean Zeta angle for the class II skeletal base group was 53° ± 4.5°. Similarly, the mean Zeta angle for the class III skeletal base group was measured at 69.8° ± 3.4°. An analysis using one-way ANOVA revealed a statistically significant variance among the three subgroup categories, further confirmed by the Bonferroni post-hoc test, which also highlighted significant differences when comparing all groups. Moreover, an independent t-test showed no significant differences in the Zeta angle mean values between sexes (Tables 2, 3, 4).

**Table 2:** Comparative assessment of the mean Zeta (ζ) angle according to malocclusion by one-way analysis of variance (ANOVA) * p-value < 0.05: significant

Groups (n = 97)	Mean ζ angle	Standard deviation	Standard error	95% confidence interval for mean		p-value
				Lower bound	Upper bound	
Class I	60.28	3.055	0.483	59.30	61.25	0.000*
Class II	53.00	4.557	0.721	51.54	54.46	
Class III	69.80	3.480	0.550	68.69	70.91	

**Table 3:** Pairwise comparison using Bonferroni post-hoc test * p value<0.05: Significant

Group pair	Mean difference	p-value
Class I vs. Class II	7.275	0.000*
Class I vs. Class III	-9.525	0.000*
Class II vs. Class III	-16.8	0.000*

**Table 4:** Comparative assessment of the mean Zeta (ζ) angle according to gender using independent Student's T-test NS: Non-significant

Groups	ζ angle (mean + standard deviation)		p-value
	Males (n = 48)	Females (n = 49)	
Class I	60.90 + 3.09	59.65 + 2.96	0.200 (NS)
Class II	52.75 + 4.63	53.25 + 4.58	0.733 (NS)
Class III	69.80 + 2.98	69.80 + 3.99	1.00 (NS)

**Figure 2:**
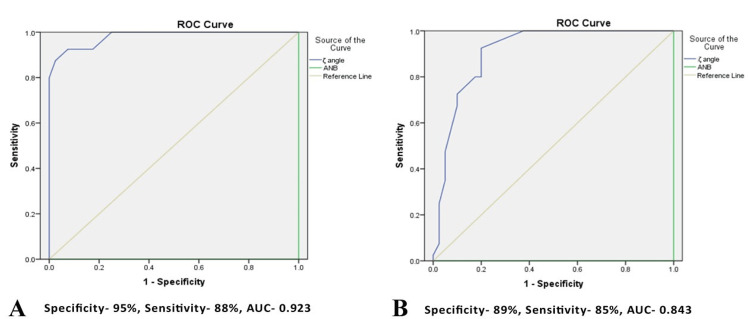
Receptor operating characteristic (ROC) curves for the Zeta angle (blue line). (A) Differentiation of Class II from Class I. (B) Differentiation of Class III from Class I. AUC (area under the curve); ANB (angle between point A, nasion, and point B)

The ROC curves demonstrated that a Zeta angle below 57.5° exhibited 80% sensitivity and 82.5% specificity in differentiating class II from the class I subset (Figure 2A). Conversely, a Zeta angle exceeding 64.5° showed 92.5% sensitivity and specificity in differentiating class III from the class I subset (Figure 2B).

According to the ROC curves, a Zeta angle cut-off value of around 57° was identified between class I and class II groups, while a cut-off value of approximately 64° was observed between class I and class III groups. These thresholds align closely with the mean values from the class I group (60.2° ± 3°), underscoring the high level of reliability. This implies that individuals with a Zeta angle between 57° and 64° truly exhibit a class I skeletal pattern. The findings further suggested that a Zeta angle less than 57° indicates a class II sagittal relation, whereas a Zeta angle greater than 64° indicates a class III sagittal relation.

## Discussion

Since its advent in 1931, cephalometrics has been considered an important diagnostic tool for the assessment of transverse, sagittal, and vertical jaw relationships. Amid skeletal base dysplasia, the sagittal relationship plays an essential role in diagnosis and treatment planning; hence, it needs to be critically evaluated. Numerous angular and linear parameters, such as the ANB angle [1], Wits appraisal [4], Beta angle [9], Yen angle [11], and W angle [12], have been proposed to assess anteroposterior dysplasia, each with their merits and inaccuracies.

The ANB angle, as introduced by Reidel and popularized by Steiner, is a widely recognized parameter for evaluating the anteroposterior relationship of the jaw [1]. However, it is important to note that this angle is not solely dependent on various factors that can influence it. Research has indicated that nasion movement during growth or jaw rotation can have a direct impact on the ANB angle. Studies have demonstrated that a 2.5° reduction occurs for every 5 mm anterior displacement, a 0.5° decrease for a 5 mm upward displacement of the nasion, and a 1° increase for a 5 mm downward displacement of the nasion. In addition, the cranial base length, cranial base inclination, and anterior facial height are contributing factors in the determination of this angle [2].

Jacobson's Wits appraisal is a widely used alternative for assessing AP severity [4]. This assessment is not reliant on cranial landmarks but instead utilizes perpendiculars drawn from points A and B on the functional occlusal plane (FOP). While the Wits appraisal remains relatively consistent regardless of age and accounts for jaw rotation, it utilizes the FOP to identify discrepancies in anteroposterior alignment. Factors, such as dental development, tooth eruption, and orthodontic treatment, can significantly affect the occlusal plane. However, accurately identifying and replicating the FOP poses challenges, and any changes to the FOP during orthodontic procedures can affect the Wits appraisal, potentially leading to an inaccurate representation of sagittal dysplasia in the jaws [6, 8].

To address the limitations associated with utilizing the occlusal plane as a reference, Chang introduced the concept of AF-BF, where the AF-BF distance is derived by perpendicular projections onto the FH plane from points A and B [16]. Nevertheless, the evaluation may be influenced by the inclination of the FH plane. In addition, a study revealed that Porion and Orbitale, commonly used in constructing the FH plane, are the least dependable reference points [17]. The Beta angle, established by Baik and Ververidou, is a widely used parameter that involves three key skeletal landmarks: Points A, B, and condylion (C) [9]. This angle was created by the intersection of a line perpendicular to the CB line at points A and AB. Although unaffected by jaw rotations, alterations in the locations of points A and B resulting from growth and orthodontic treatments may influence the angle [2]. Moreover, difficulties emerge in precisely identifying the center of the condyle while guaranteeing the accuracy, consistency, and estimation of this parameter [10].

To address the limitations of the Beta angle, Neela introduced the Yen angle, which is determined by points S, M, and G (midpoint of the largest circle tangent to the internal surfaces of the mandibular symphysis) [11]. Despite utilizing reliable landmarks, the Yen angle may be affected by jaw rotation, leading to potential inaccuracies [2]. However, the W angle maximizes the same points as the Yen angle, but the angle is measured between a perpendicular drop from point M to the SG and MG lines [12]. Although unaffected by jaw rotations, it depends on point S, which has been deemed unstable by numerous studies [13]. Li et al. (2022) introduced G triangle analysis as a method for evaluating sagittal relationships [18]. In contrast to the ANB angle, this approach does not incorporate the nasion point into the triangle construction. Instead, it utilizes points A and B to examine the relationship between the maxilla and the mandible. It is important to note that points A and B are susceptible to changes owing to growth rotations and orthodontic interventions, as previously mentioned [2].

The majority of the parameters introduced for assessing anteroposterior dysplasia seem to be influenced by the patient’s age, growth, rotation of the apical bases, poor accuracy, and reproducibility of the chosen landmarks or orthodontic treatment mechanics. Thus, to overcome the limitations of the aforementioned parameters, the Zeta angle was developed. The Zeta angle uses three stable skeletal landmarks, namely, point Pt, point M, and point Pm, eliminating the use of unstable landmarks, cranial reference plane, or occlusal plane.

The Zeta angle utilizes anatomical landmarks on the skeleton: points M and Pm, serving as representatives of the maxilla and mandible, respectively. Point M, attributed to Nanda and Merrill [7], was subsequently identified by Braun as the midpoint of the premaxilla, symbolizing the center of the largest circle that touches the anterior, superior, and palatal surfaces of the premaxilla. In contrast to points A and B, point M remains unaffected by local restructuring resulting from dental shifts or orthodontic interventions. Furthermore, it can serve as a means to analyze the growth trajectory of the maxilla, thus highlighting the reliability of point M during periods of active growth [19]. The suprapogonion (Pm) point is located at a pivotal stress point and serves as the location of a reversal line. Implant research suggests its stability as an unchanging bone in the chin, offering a consistent reference point in the mandible [20].

The pterygoid point, denoted as the Pt point, serves as a locus of energy owing to innervation of the maxillary nerve [21]. Pt denotes the nearest location and alignment to the center of minimal growth, thereby facilitating its utility for sequential comparisons. In the context of superimposing tracings on a polar grid, Pt, as highlighted by Ricketts, signifies a pivotal aspect of growth that emanates from its specific placement. This assertion is supported by Brodie, who put forth thoughts regarding the enduring stability of the pterygopalatine fossa [22].

The stability of the Zeta angle is maintained when the jaws undergo rotation owing to its geometric properties. The rationale behind this occurrence is attributed to the consistent alignment of the perpendicular originating from point M with the Pt-Pm line because of the rotational movements of the jaws. Furthermore, as the M-Pm line undergoes rotation in the same direction, the zeta angle remains consistently stable. Therefore, the measurement of the Zeta angle proves to be advantageous in the evaluation of sagittal dysplasia linked with upward or backward jaw movements, as well as during the transitional phase of vertical facial development. Another benefit of utilizing this angle is its utility in monitoring treatment progress, as it reflects genuine alterations in sagittal alignment that may arise from natural growth processes or orthodontic interventions.

Nevertheless, precise delineation of the premaxilla and determination of its midpoint can pose challenges; therefore, acquiring a high-quality cephalogram is imperative to facilitate the tracing of the premaxillary outline and identification of its central point. The Zeta angle alone is inadequate for determining the prognathic or retrognathic nature of the jaw in cases with class II and class III apical bases. Therefore, clinicians should recognize the need for additional cephalometric information.

In the literature, there is a plethora of parameters aimed at delineating sagittal dysplasia through cephalometrics; however, discernible limitations are evident. Consequently, it is imperative for clinicians to exercise prudence and caution regarding their use. The Zeta angle serves to bolster the existing cephalometric parameters in evaluating sagittal dysplasia, thereby enhancing the accuracy of diagnosis and treatment planning for orthodontic patients.

Limitations

The current research faced a significant constraint owing to its retrospective nature and focused on subjects of North Indian descent. Consequently, future prospective studies should be conducted with more extensive cohorts of individuals from diverse ethnic backgrounds.

## Conclusions

Formerly acknowledged cephalometric measurements for evaluating sagittal dysplasia may lead to inaccuracies; therefore, a novel angle known as the Zeta angle was introduced as an additional tool for consistent identification of sagittal jaw relationships. Individuals exhibiting a Zeta angle ranging from 57° to 64° typically present with a class I skeletal jaw pattern, while a Zeta angle below 57° suggests a class II skeletal jaw pattern and a Zeta angle exceeding 64° indicates a class III skeletal jaw pattern. Gender-based analysis revealed a lack of statistical significance in average Zeta angle values.
